# Fluoxetine Decreases the Proliferation and Adipogenic Differentiation of Human Adipose-Derived Stem Cells

**DOI:** 10.3390/ijms160716655

**Published:** 2015-07-22

**Authors:** Bo Kyung Sun, Ji Hye Kim, Joon-Seok Choi, Sung-Joo Hwang, Jong-Hyuk Sung

**Affiliations:** 1 College of Pharmacy, Yonsei University, Incheon 406-840, Korea; E-Mails: bksun2011@gmail.com (B.K.S.); wisdom-ks@hanmail.net (J.H.K.); 2 STEMORE Co., Ltd., Incheon 406-840, Korea; 3 College of Pharmacy, Catholic University of Daegu, Daegu 712-702, Korea; E-Mail: joonschoi@naver.com

**Keywords:** adipose-derived stem cells, fluoxetine, autophagy, proliferation, adipogenic differentiation

## Abstract

Fluoxetine was originally developed as an antidepressant, but it has also been used to treat obesity. Although the anti-appetite effect of fluoxetine is well-documented, its potential effects on human adipose-derived stem cells (ASCs) or mature adipocytes have not been investigated. Therefore, we investigated the mechanisms underlying the inhibitory effects of fluoxetine on the proliferation of ASCs. We also investigated its inhibitory effect on adipogenic differentiation. Fluoxetine significantly decreased ASC proliferation, and signal transduction PCR array analysis showed that it increased expression of autophagy-related genes. In addition, fluoxetine up-regulated SQSTM1 and LC3B protein expression as detected by western blotting and immunofluorescence. The autophagy inhibitor, 3-methyladenine (3-MA), significantly attenuated fluoxetine-mediated effects on ASC proliferation and SQSTM1/LC3B expression. In addition, 3-MA decreased the mRNA expression of two autophagy-related genes, beclin-1 and Atg7, in ASCs. Fluoxetine also significantly inhibited lipid accumulation and down-regulated the levels of PPAR-γ and C/EBP-α in ASCs. Collectively, these results indicate that fluoxetine decreases ASC proliferation and adipogenic differentiation. This is the first *in vitro* evidence that fluoxetine can reduce fat accumulation by inhibiting ASC proliferation and differentiation.

## 1. Introduction

Adipose-derived stem cells (ASCs) exist in adipose tissue and can be isolated from lipoaspirates during elective surgical procedures. ASCs can be obtained in large quantities using a simple isolation procedure, and have the potential for utilization in tissue repair and regeneration [1,2,3]. For example, we demonstrated that ASCs exhibited wound-healing, anti-wrinkle and hair-regenerative potential through building-block functions and paracrine effects [4,5,6,7]. In adipose tissue, ASCs are found in perivascular regions and can be induced to differentiate into mature adipocytes by environmental trophic factors [8,9,10]. Although it is unclear whether ASCs can differentiate into endothelial cells *in vivo*, ASCs recruit endothelial cells to form microvessels in adipose tissue [11,12,13]. Therefore, adipocyte differentiation of ASCs and angiogenesis by endothelial cells collectively contribute to fat accumulation.

Obesity is an excess of body weight that is associated with an increased accumulation of adipose tissue. It is a risk factor for chronic diseases such as hypertension, coronary heart disease, stroke, hyperlipidemia, and diabetes. Current strategies for obesity management include diet, exercise, drug therapy, and bariatric surgery. Current anti-obesity drugs are designed to suppress appetite and reduce fat absorption [14,15]. Fluoxetine was originally developed as an antidepressant and it acts as a selective serotonin reuptake inhibitor [16,17]. Of interest, it has been reported that fluoxetine induced the neuronal differentiation of adult stem or progenitor cells *in vivo* [18,19]. Clinical studies demonstrated that serotonergic drugs specifically reduced appetite prior to and following the consumption of fixed caloric loads, and reduced pre-meal appetite and caloric intake [20,21,22]. Although the anti-appetite effect of fluoxetine is well-documented, its potential effects on ASCs or mature adipocytes have not been investigated.

Autophagy is the basic catabolic mechanism that facilitates degradation of unnecessary or dysfunctional cellular components through the actions of lysosomes [23,24]. During this process, targeted cytoplasmic constituents are isolated from the rest of the cell within a membrane vesicle known as an autophagosome. Autophagy is an adaptive response to stress that promotes survival; however, under other circumstances it can promote cell death and morbidity. Prolonged autophagy leads to a high turnover rate of proteins and different organelles, which may cause cell death. Interestingly, it was reported that antidepressants such as maprotiline and fluoxetine induced autophagic cell death in Burkitt’s lymphoma [25]. In addition, other antidepressant, FKBP51, primed autophagy pathways in lymphocytes [26]. However, the effects of fluoxetine on autophagy or cell death have not been investigated in ASCs. In addition, activation of autophagy suppressed the adipogenic differentiation of ASCs [27,28]. Therefore, the present study investigated: (1) whether fluoxetine induced autophagic cell death in ASCs; (2) the molecular mechanisms and/or signal pathways involved in autophagic cell death; and (3) whether fluoxetine inhibited adipogenic differentiation of ASCs.

## 2. Results and Discussion

### 2.1. Fluoxetine Decreased ASC Proliferation

**Figure 1:**
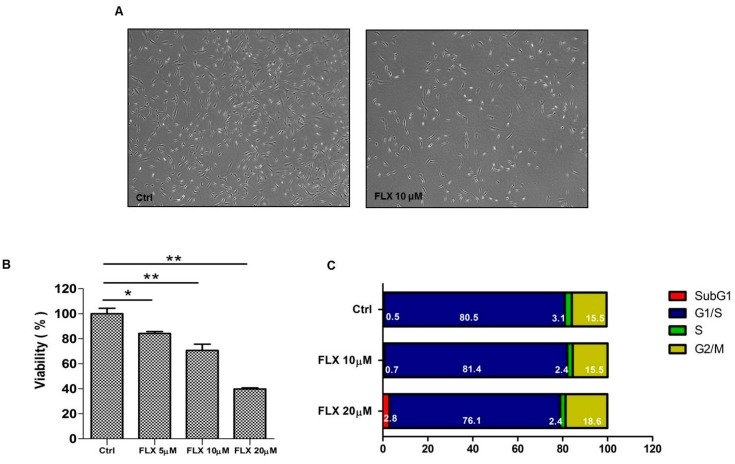
Fluoxetine decreased the proliferation of ASCs in the CCK-8 assay. (**A**) Morphology of ASCs at 48 h after fluoxetine treatment, 40×; (**B**) Fluoxetinen (5–20 μM) significantly decreased the proliferation of ASCs in CCK-8 assay. All values are presented as a mean ± STD (*n* = 3). * *p* < 0.05, ** *p* < 0.01; (**C**) Cell cycle analysis indicated that 20 µM fluoxetine increased the cell cycle arrest (SubG) and induced apoptosis in ASCs.

Proliferation of ASCs was measured at 48h after fluoxetine treatment. Figure 1 shows that 5-20 μM fluoxetine significantly decreased the proliferation of ASCs in a dose-dependent manner (Figure 1A,B, *p* < 0.05). To determine what phases of the cell cycle were affected by fluoxetine treatment, altered cell cycle was measured using flow cytometry. Fluoxetine did not alter cell cycle in 10 µM, but increased the cell cycle arrest (*i.e.*, SubG1) at 20 µM concentration (Figure 1C). This indicates that fluoxetine induces apoptosis of ASCs at high concentration.

### 2.2. Fluoxetine Induced Autophagy

**Figure 2:**
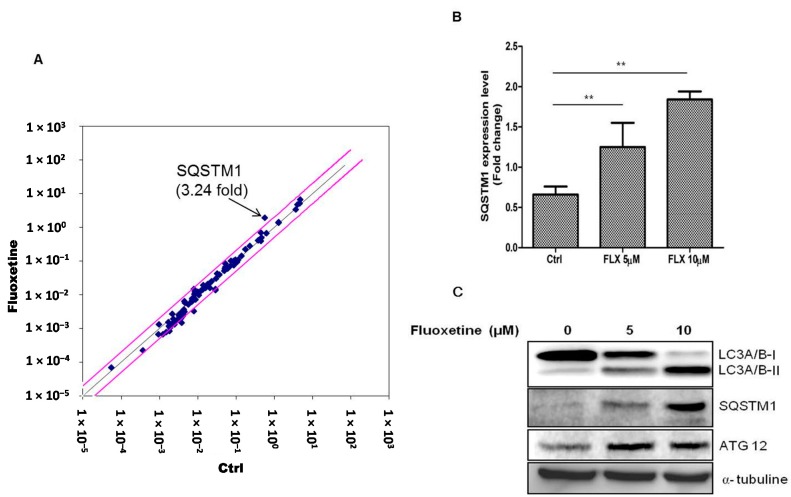
Fluoxetine induced autophagy. (**A**) Signaling pathway involved in the ASC suppression by fluoxetine was determined using RT^2^ profiler™ PCR array, and fluoxetine treatment for 4 h increased the mRNA level of SQSTM1 in ASCs; (**B**) SQSTM1 mRNA upregulation was confirmed by qPCR (Figure 2B, *p* < 0.01). GAPDH was used for normalization. All values are presented as a mean ± STD (*n* = 3). ** *p* < 0.01. (**C**) Protein level of autophagy-related genes was measured using western blotting at 48 h after fluoxetine treatment. LC3A (Cell Signaling Technology, #12741) expression decreased, while LC3B levels increased. In addition, SQSTM1 (Cell Signaling Technology, #5114) and ATG12 (Cell Signaling Technology, #4180) levels were up-regulated.

Because fluoxetine decreased the proliferation, we examined the signaling pathways involved in fluoxetine-mediated suppression of ASC proliferation using RT^2^ profiler™ PCR array (signal transduction pathway finder). The results are listed in Supplementary Table S1. Fluoxetine treatment for 4 h increased the mRNA level of SQSTM1 in ASCs (Figure 2A) and we therefore hypothesized that fluoxetine induced autophagic cell death in these cells. Real-time PCR (qPCR) assay also identified the increased levels of SQSTM1 mRNA (Figure 2B, *p* < 0.01). In addition, we measured the levels of the proteins encoded by autophagy-related genes using western blotting (at 48 h after fluoxetine treatment). LC3A/B-I expression decreased, while LC3A/B-II increased. In addition, SQSTM1 and ATG12 levels were up-regulated (Figure 2C).

### 2.3. Fluoxetine Increased Autophagosome Formation in ASCs

**Figure 3:**
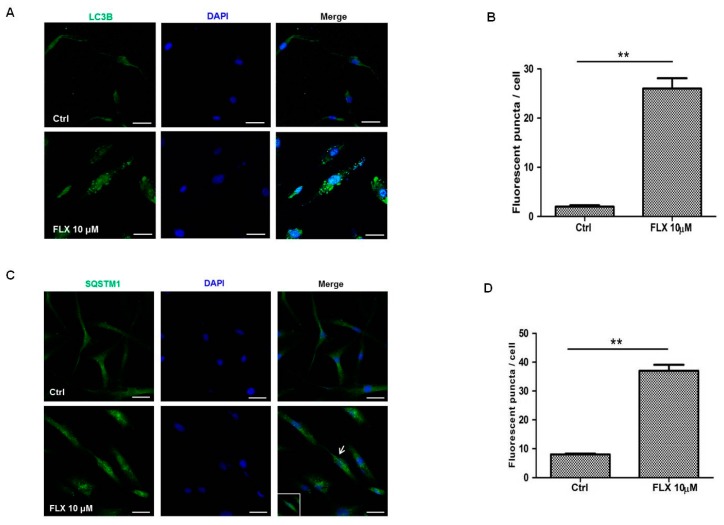
Fluoxetine increased autophagosome formation in ASCs. (**A**) Punctate expression of LC3B (Santa Cruz Biotech, sc-28266) is common feature of autophagy, Scale bars = 20 μm; (**B**) Fluorescence puncta number was measured, and fluoxetine significantly increased the punctate fluorescence signal in ASCs; (**C**) Although punctate expression of SQSTM1 (arrow, Santa Cruz Biotech, sc-28359) is not as clear as that of LC3B, SQSTM1 staining also formed punctate-like structures. Scale bar = 20 μm; (**D**) Fluorescence puncta number was measured, and fluoxetine significantly increased the fluorescence signal of SQSTM1 in ASCs. All values are presented as a mean ± STD (*n* = 3). ** *p* < 0.01.

As western blotting indicated that fluoxetine increased LC3B and SQSTM1 protein levels, we further examined whether fluoxetine increased autophagosome formation in ASCs using immunofluorescence. Punctate expression of LC3B is a common feature of autophagy, and fluoxetine significantly increased this punctate signal in ASCs (Figure 3A,B, *p* < 0.01). Although punctate expression of SQSTM1 was not as clear as that of LC3B, fluoxetine significantly increased the SQSTM1 fluorescence signal in ASCs (Figure 3C,D, *p* < 0.01).

### 2.4. Autophagy Inhibition Study

**Figure 4:**
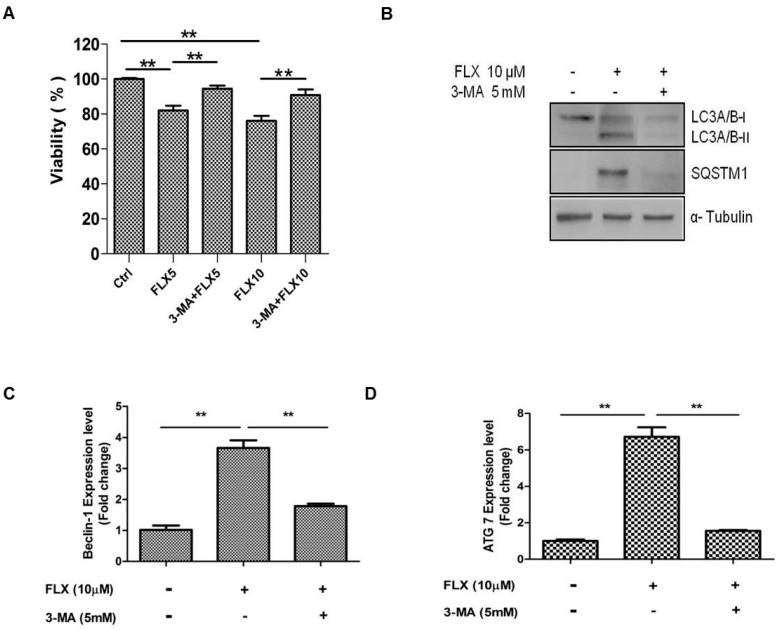
Autophagy inhibition study. (**A**) 3-Methyladenine (3-MA, an autophagy inhibitor) significantly attenuated fluoxetine-induced suppression of ASC viability; (**B**) 3-MA down-regulated the fluoxetine-induced effects on the protein levels of SQSTM1 and LC3B detected by western blotting; (**C**,**D**) 3-MA significantly down-regulated the mRNA levels of the autophagy-related genes, beclin-1 (**C**) and Atg7 (**D**), detected by quantitative real-time PCR. All values are presented as a mean ± STD (*n* = 3). ** *p* < 0.01.

In a previous study, fluoxetine-induced autophagy was attenuated by 3-MA, and we hypothesized that 3-MA can inhibit fluoxetine-induced autophagy [25]. As expected, 3-MA, the inhibitor of autophagy significantly attenuated fluoxetine-mediated suppression of ASC viability (Figure 4A, *p* < 0.05). 3-MA also down-regulated the fluoxetine-induced increase in the SQSTM1 mRNA level (as observed by qPCR, data not shown). 3-MA also attenuated the SQSTM1 and LC3A/B-I protein levels, as observed by western blotting (Figure 4B). Furthermore, 3MA significantly down-regulated the mRNA levels of autophagy-related genes such as beclin-1 (Figure 4C, *p* < 0.01) and Atg7 (Figure 4D, *p* < 0.01), detected by qPCR.

### 2.5. Fluoxetine Decreased the Adipognic Differentiation of ASCs

**Figure 5:**
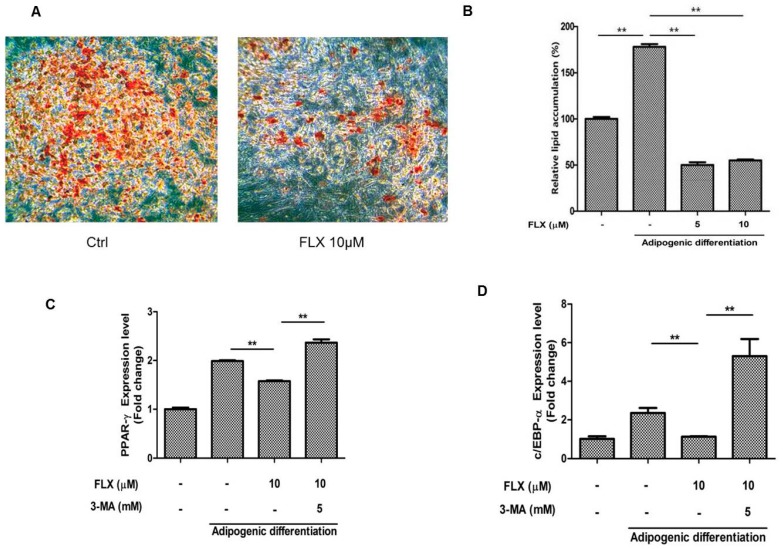
Fluoxetine decreased the adipogenic differentiation of ASCs. (**A**) ASCs were cultured in adipocyte induction medium for two weeks, stained using Oil Red O, and microscopic images are taken, 40×; (**B**) Fluoxetine significantly reduced the lipid accumulation in adipocyte induction medium; (**C**) Fluoxetine decreased the levels of adipogenic differentiation markers, peroxisome proliferator-activated receptor-γ (PPAR-γ) at day 7, but the autophagy inhibitor 3-MA significantly attenuated the mRNA levels of PPAR-γ; (**D**) Fluoxetine decreased the levels of adipogenic differentiation markers, CCAAT-enhancer-binding proteins-α (C/EBP-α) at day 7, but 3-MA significantly reversed the effect of fluoxetine on C/EBP-α mRNA levels, as detected by quantitative real-time PCR. GAPDH was used for normalization. All values are presented as a mean ± STD (*n* = 3). ** *p* < 0.01.

It has been reported that activation of autophagy suppresses the adipogenic differentiation of ASCs [27,28]. Therefore, we examined whether fluoxetine decreased the adipogenic differentiation of ASCs. As expected, fluoxetine slightly decreased the level of lipid droplets observed using Oil Red O staining (Figure 5A,B, *p* < 0.01). Fluoxetine decreased the levels of adipogenic differentiation markers, peroxisome proliferator-activated receptor-γ (PPAR-γ) at day 7, but the autophagy inhibitor 3-MA significantly attenuated the mRNA levels of PPAR-γ (Figure 5C, *p* < 0.01). In addition, fluoxetine decreased the levels of adipogenic differentiation markers, CCAAT-enhancer-binding proteins-α (C/EBP-α) at day 7, but an autophagy inhibitor 3-MA significantly attenuated the mRNA levels of C/EBP-α, as detected by quantitative real-time PCR (Figure 5D, *p* < 0.01).

### 2.6. Discussion

In the present study, we primarily investigated the inhibitory effect of fluoxetine on ASC proliferation and the mechanism underlying this effect. We also investigated its inhibition of adipogenic differentiation. Fluoxetine significantly decreased the proliferation of ASCs, and increased the levels of SQSTM1 in a signal transduction PCR array. In addition, fluoxetine up-regulated the protein expression of SQSTM1 and LC3B detected by western blotting, and increased their punctate immunofluorescent signals. Autophagy inhibitor, 3-MA, significantly attenuated fluoxetine-induced SQSTM1 and LC3B expression. Furthermore, 3-MA decreased the mRNA levels of other autophagy-related genes in ASCs, such as beclin-1 and Atg7. On the other hand, fluoxetine significantly inhibited lipid accumulation in ASCs and down-regulated their expression of PPAR-γ and C/EBP-α in adipocyte induction medium. Collectively, these results indicated that fluoxetine decreased the proliferation and adipogenic differentiation of ASCs. This is the first *in vitro* evidence that fluoxetine might inhibit fat accumulation by these mechanisms.

Blood or plasma fluoxetine concentrations are reportedly 0.15–1.5 μM in persons taking this drug for its antidepressant effects, and 2.7–10 μM in survivors of acute overdosage [21,29]. Although local concentration of fluoxetine has not been measured in the adipose tissue of patients who take fluoxetine for anti-obesity, fluoxetine is hydrophobic and may accumulate in this tissue. Therefore, accumulated fluoxetine in adipose tissue may inhibit the proliferation and adipogenic differentiation of ASCs, resulting in decreased fat accumulation.

Autophagy is a dynamic cellular event that is involved in the degradation of long-lived proteins and organelles within cells. Biochemical methods such as western blot and immunohistochemistry have been increasingly used to measure autophagic proteins. Measurement of the total amount of LC3, the mammalian homologue of the autophagy-related Atg8 in yeast, is a very useful and commonly used tool in autophagy studies [30]. LC3 is a soluble protein with a molecular mass of approximately 17 kDa that is distributed ubiquitously in mammalian tissues and cultured cells. A cytosolic form of LC3 (LC3I) is conjugated to phosphatidylethanolamine to form LC3-phosphatidylethanolamine conjugate (LC3II), which is recruited to autophagosomal membranes. Thus, lysosomal turnover of the autophagosomal marker LC3II reflects autophagic activity, and detection of LC3 by immunoblotting or immunofluorescence has become a reliable method for monitoring autophagy and autophagy-related processes, including autophagic cell death [30]. In the present study, western blotting indicated that fluoxetine decreased the LC3A/B-I level, while increasing the LC3A/B-II level (Figure 2C). In addition, fluoxetine increased the number of puncta signal of LC3B identified by immunofluorescence studies (Figure 3A), which collectively indicated that fluoxetine induced autophagic cell death in ASCs.

As described above, fluoxetine has different effects on neuronal progenitor cells (NPCs) and bone-marrow-derived mesenchymal stem cells (BMSCs). For example, fluoxetine increased the proliferation of embryo-derived NPCs through up-regulating GSK-3β/β-catenin signaling and this is linked with serotonin receptor activation [19]. In addition, fluoxetine induced the neuronal differentiation of BMSCs and increased the number of dendrites [18]. However, in the present study, fluoxetine decreased the survival of ASCs and suppressed the adipogenic differentiation of ASCs. We did not further investigate the underlying mechanism of cell-type dependent difference, but there is supporting evidence that induction of autophagy by fluoxetine contributed to the cell death in Burkitt’s lymphoma [25].

In summary, Fluoxetine decreased ASC proliferation, and increased expression of autophagy-related genes such as SQSTM1 and LC3B. Fluoxetine also inhibited lipid accumulation and down-regulated the levels of PPAR-γ and C/EBP-α in ASCs. This is the first *in vitro* evidence that fluoxetine can reduce fat accumulation by inhibiting ASC proliferation and differentiation. In addition to appetite suppression, our results suggest that inhibition of ASC proliferation and differentiation may partly contribute to the weight loss associated with fluoxetine treatment.

## 3. Experimental Section

### 3.1. Materials

Fluoxetine and 3-methyladenine (3-MA) were obtained from Sigma-Aldrich (Saint Louis, MO, USA). Antibodies recognizing Akt (1:3000), phospho-Akt (1:2000), phospho-mTOR (1:2000), and LC3A/B (1:2000), SQSTM1 (1:100 or 1:1000) and ATG12 (1:1000) were purchased from Cell Signaling Technology (Danvers, MA, USA). α-Tubulin (1:10,000) and LC3B (1:1000) were purchased from Santa Cruz Biotechnology (Santa Cruz, CA, USA). Horseradish-peroxidase (HRP)-conjugated secondary mouse antibody (1:10,000) and HRP-conjugated secondary rabbit antibody (1:1000) were purchased from Cell Signaling Technology. Human signal transduction pathwayfinder RT^2^ profiler PCR Array (PAHS-041ZD) was obtained from SABiosciences (Qiagen, Hilden, Germany).

### 3.2. Cell Culture

ASCs were isolated as described previously [3], and cultured in minimum essential medium alpha (α-MEM; Hyclone, Thermo Scientific, Logan, UT, USA) with 10% fetal bovine serum (FBS; GIBCO, Invitrogen, Carlsbad, CA, USA), and 1% penicillin and streptomycin (GIBCO). ASCs were maintained at 37 °C in a humidified 5% CO_2_ incubator. Characterization of ASCs was performed using cell surface markers such as CD34, CD73, CD90, and CD105. Potential for multiple differentiation also has been confirmed [3].

### 3.3. Cell Proliferation Assay

ASCs were seeded in a 48-well plate at a density of 5 × 10^3^ cells/well, and attached in complete medium. Then, the medium was replaced with α-MEM containing 0.2% FBS. The following day, cells were treated with fluoxetine (5, 10, and 20 μM) for 48 h, with or without 3-MA (5 mM). Incubating medium was removed and cell numbers were counted using the CCK-8 assay kit (Dojindo, Rockville, MD, USA). Cells were treated with 10% CCK-8 solution in medium for 2 h at 37 °C in a humidified 5% CO_2_ incubator prior to measurement of the absorbance at 450 nm using a microplate reader (TECAN, Gordig, Austria).

### 3.4. Adipocyte Differentiation

ASCs were seeded in a 12-well plate at the density of 4 × 10^4^ cells in the complete medium. When ASCs became confluent, culture medium was replaced with preadipocyte growth medium-2 (PGM-2, Lonza, Walkersville, MD, USA). Then, cells were treated with fluoxetine for 7–14 days. Oil Red O staining was used to assess lipid accumulation in ASCs [31].

### 3.5. Oil Red O Staining

ASCs were washed with PBS and fixed with 10% formalin for 1 h. After fixing, cells were washed with PBS and stained with Oil Red O solution (1% Oil Red O in isopropanol:double-distilled water at a ratio of 6:4, mixed immediately before use) for 2 h. The stained cells were washed twice with distilled water and examined microscopically. For quantification of Oil Red O, cells were incubated with isopropanol for 30 min, and the color in the supernatant of the decolorized cells was measured by the absorbance at 492 nm with a micro-plate reader (TECAN).

### 3.6. Western Blotting

Cells were rinsed twice with ice-cold PBS and then extracted with NP-40 lysis buffer (0.5 % NP-40, 50  mM Tris-HCl pH 7.4, 120 mM NaCl, 25 mM NaF, 25 mM glycerol phosphate, 1 mM EDTA, and 5 mM EGTA) containing a complete protease inhibitor cocktail tablet (Roche, Basel, Switzerland). Lysates were collected and centrifuged at 15, 000× *g* for 15 min at 4  °C. The samples were separated by sodium dodecyl sulfate-polyacrylamide gel electrophoresis and transferred to polyvinylidene fluoride membrane (Millipore, Bedford, MA, USA). The membrane was blocked with 3% bovine serum albumin for 1 h at room temperature and then incubated with primary antibody overnight at 4 °C. The following day, the membrane was washed with Tris-buffered saline containing 0.1% Tween 20 and then incubated with HRP-conjugated secondary antibody for 1 h. The protein bands were detected by an enhanced chemiluminescence western blotting analysis system (Thermo Scientific, Waltham, MA, USA).

### 3.7. Immunostaining of SQSTM1 and LC3B

ASCs were seeded on circular glass cover-slips. The following day, cells were incubated with culture medium (0.2% FBS) containing 10 μM fluoxetine for 48 h. Then, the cells were fixed with 4% paraformaldehyde for 15 min and permeabilized using 0.5% PBS-T for 5 min. After washing with 0.1% PBS-T, cells were blocked using blocking solution (10% FBS and 0.5% gelatin in PBS) for 30 min. Primary antibody (SQSTM1 and LC3B) was diluted to a concentration of 1:200 and secondary antibody (FITC, Invitrogen) to 1:200. For nuclear staining, cells were stained with DAPI (Sigma, D9542). ASCs were counterstained with 0.1 µM DAPI for 5 min and washed with PBS. Fluorescence signals were detected using a fluorescence confocal microscope (Carl Zeiss, Oberkochen, Germany).

### 3.8. RNA Isolation and qPCR

Total cellular RNA was extracted using an RNA prep kit (RNeasy™, Qiagen) and quantificated using Nanodrop (Thermo Scientific). Total RNA was reverse-transcribed with a cDNA synthesis kit (A2500, Promega, Madison, WI, USA), and cDNA was synthesized from 500 ng of total RNA using 10,000 U reverse transcriptase and 50 ng/mL oligo(dT) primers (RT conditions were 25 °C for 5 min, 42 °C for 60 min, 70 °C for 15 min). Thermal cycling consisted of an initial denaturation at 95 °C for 5 min, followed by 35 cycles of 95 °C for 30 s, 56 °C for 20 s, and 72 °C for 40 s, and terminated by a final extension at 72 °C for 5 min. qPCR reactions were performed on a Step One Plus Real-Time PCR system (Applied Biosystems, Invitrogen) using SYBR Green PCR Master Mix (Takara Bio, Inc., Otsu, Japan). The level of GAPDH was also quantified for sample standardization. Analysis of fold change was calculated using the Δ*C*_t_ value.

### 3.9. PCR Array

The human signal transduction pathway finder RT^2^ profiler PCR Array (PAHS-041ZD) was obtained from SABiosciences (Qiagen). Cells were seeded on 60 mm dishes at a density of 2.5 × 10^5^ cells/well and cultured in complete medium. After starvation, fluoxetine was treated for 4 h in α-MEM containing 0.2% FBS. Total RNA was harvested with an RNA prep kit (RNeasy™, Qiagen) and cDNA was synthesized using reverse transcriptase as described above. Gene expression was detected by the RT^2^ profiler PCR array kit according to the manufacturer’s instructions.

### 3.10. Statistical Analysis

All data were representative of triplicate, independent experiments. The statistical significance of differences among groups was tested using a Student’s *t*-test. *p* < 0.05 or *p* < 0.01 was considered to be significant.

## 4. Conclusions

This study provides evidence that fluoxetine decreases the proliferation of ASCs through autophagy. Fluoxetine also decreases the adipogenic differentiation of ASCs. In addition to appetite suppression, our results suggest that inhibition of ASC proliferation and adipogenic differentiation may partly contribute to the weight loss associated with fluoxetine treatment.

## Supplementary Materials

Supplementary materials can be found at http://www.mdpi.com/1422-0067/16/07/16655/s1.
